# Maternal and Neonatal Outcomes in Very Young Adolescent Pregnancies: A Single-Centre Retrospective Observational Study in Brașov, Romania

**DOI:** 10.3390/healthcare14040499

**Published:** 2026-02-14

**Authors:** Abdul Jabar Khudor, Marius Alexandru Moga, Oana Gabriela Dimienescu, Andrada Camelia Nicolau, Natalia (Ciobanu) Vasilachi, Mircea Daniel Hogea

**Affiliations:** 1 Medicine PhD School, Transilvania University of Brasov, 500036 Brașov, Romania; 2 Faculty of Medicine, Transilvania University of Brasov, 500036 Brașov, Romania

**Keywords:** adolescent, pregnancy, teenage, obstetrics, complications, maternal outcomes, neonatal outcomes, Romania, low birth weight, preeclampsia

## Abstract

Background: Adolescent pregnancies remain associated with increased maternal and neonatal morbidity. This study describes the clinical and demographic characteristics of very young pregnant adolescents in Brașov County, Romania. Methods: We conducted a retrospective observational study of 1322 singleton deliveries to adolescents aged 12–16 years at Clinical Hospital “Dr. I.A. Sbârcea”, Brașov (2018–2024). Descriptive statistics, Chi-square tests, *t*-tests, and correlation analyses were performed. No multivariable adjustment was applied. Results: Mean maternal age was 15.3 ± 0.8 years; 82.8% were from rural areas; 76% were primigravida. Cesarean section rate was 31.5%. Maternal complications included anemia (45%), postpartum hemorrhage (29.4%), preeclampsia (8%), and urogenital infections (28%). Among neonates, 33.7% had low birth weight, 18% were preterm, and 32.4% had APGAR scores < 7 at 1 min. Adolescents aged 12–14 years had lower postpartum hemoglobin (8.72 vs. 10.42 g/dL, *p* < 0.001) and higher rates of APGAR < 7 at 1 min (51.4% vs. 28.8%, *p* < 0.001) compared to those aged 15–16 years. Rural residence was associated with higher anemia rates (46.8% vs. 36.4%, *p* = 0.003) and inadequate prenatal care (40.0% vs. 23.7%, *p* < 0.001). Conclusions: This single-centre cohort shows high rates of obstetric complications and adverse neonatal outcomes among very young adolescents, with observed differences by age and residence. These descriptive findings may inform further research into risk factors and preventive interventions for adolescent pregnancies in Romania.

## 1. Introduction

Adolescent pregnancies represent a significant global public health challenge, with implications for maternal and neonatal health outcomes, as well as long-term social and economic consequences for both mother and child. According to the World Health Organization (WHO), adolescent pregnancy is defined as pregnancy in girls aged 10–19 years, with approximately 21 million pregnancies occurring annually worldwide [1]. These pregnancies are associated with increased risks of maternal complications including preeclampsia, eclampsia, puerperal endometritis, systemic infections, low birth weight (LBW), and preterm birth [2,3,4,5,6]. The biological immaturity of adolescent mothers, combined with inadequate prenatal care and socioeconomic disadvantages, has been associated with adverse outcomes in previous research [7,8,9,10,11].

Substantial inequities characterize adolescent pregnancy outcomes based on socioeconomic status, educational attainment, geographic location, and access to healthcare services. Adolescents from lower socioeconomic backgrounds, those with limited education, and those residing in rural areas shows higher rates of pregnancy complications and poor birth outcomes [12,13,14,15,16,17,18]. Limited access to contraceptive services, comprehensive sexuality education, and quality prenatal care further exacerbate these modification [19,20,21]. Additionally, pregnant adolescents have higher risks of mental health problems, social isolation, and reduced educational and economic opportunities [10,22,23,24,25]. Complications during delivery is associated with high mortality rate among adolescent girls globally, particularly in resource-limited places [26,27,28].

Romania maintains one of the highest adolescent pregnancy rates within the European Union, with approximately 16,000–18,000 births to adolescent mothers annually [19,29,30,31]. National statistics indicate that over 80% of adolescent pregnancies occur in rural areas, where access to reproductive health services and comprehensive sexuality education remains significantly limited [32,33]. The adolescent birth rate in Romania exceed average, pointing out persistent challenges in healthcare accessibility, educational opportunities, and socioeconomic development, particularly in rural and marginalized communities [30,32]. Despite national efforts to reduce adolescent pregnancy rates, significant regional variations persist, with certain counties reporting disproportionately high incidences [2,34].

Despite Romania’s high adolescent pregnancy rates, there are limited published data characterizing obstetric and neonatal outcomes specifically among very young adolescents (aged 12–16 years) in the central region of the country. This age group represents a distinct population with unique biological, psychological, and social vulnerabilities. Pregnancies in very young adolescents (under 16 years) are associated with particularly high risks due to incomplete physical maturation, active pubertal changes, and limited psychosocial maturity. Additionally, this population often faces compounding social vulnerabilities including limited education, poverty, early school dropout, and inadequate access to reproductive health services.

Brașov County represents a typical mixed urban–rural area with both tertiary referral services and underserved communities, yet no comprehensive analysis of pregnancy outcomes in this vulnerable age group has been published from this region. We chose a single-centre design to ensure data consistency and quality, as all cases were managed according to standardized institutional protocols and documented in a unified electronic medical record system. As the primary referral centre for Brașov County, this hospital manages most adolescent deliveries in the region, including both uncomplicated cases from the community and high-risk referrals. While this design limits generalizability, it provides detailed clinical data unavailable in national registries and allows for future prospective studies and quality improvement initiatives at the institutional level. This study aimed to describe the demographic and clinical characteristics of pregnancies in adolescents aged 12–16 years delivering at a single tertiary centre in Brașov between 2018 and 2024; to compare maternal and neonatal outcomes between younger (12–14 years) and older (15–16 years) adolescents; to examine differences in outcomes between rural and urban residents; and to describe temporal trends in adolescent deliveries over the study period. These descriptive findings may help generate hypotheses for future analytical studies and inform the design of targeted healthcare services for pregnant adolescents in similar settings.

## 2. Materials and Methods

We conducted a retrospective observational study at the Clinical Hospital of Obstetrics and Gynecology “Dr. I.A. Sbârcea”, Brașov, Romania, between the period January 2018 to December 2024. This tertiary referral center serves Brașov County and surrounding areas, managing approximately 4000–4500 deliveries annually, including high-risk pregnancies. As a referral centre, the hospital receives transfers of complicated cases from primary and secondary healthcare facilities, which may result in an over-representation of high-risk pregnancies compared to population-based samples.

### 2.1. Study Population and Selection Criteria

The study included all consecutive adolescent pregnancies meeting the following inclusion criteria: maternal age between 12 and 16 years at the time of delivery, singleton pregnancies, delivery at the study hospital during the specified period, complete medical records available for analysis, and documented informed consent for data utilization as per Romanian healthcare legislation. Exclusion criteria were maternal age > 16 years at delivery, multiple pregnancies (twins, triplets), pregnancies complicated by major fetal malformations diagnosed prenatally, incomplete or illegible medical documentation, and explicit refusal of consent for study participation. Multiple pregnancies were excluded because they carry different risks (preterm birth, low birth weight, pregnancy complications) that could confound comparisons with singleton pregnancies, and the small number of multiples (n = 18, 1.3% of eligible pregnancies) would not allow for meaningful subgroup analysis. Major congenital malformations were excluded (n = 23, 1.7% of eligible pregnancies) because neonatal outcomes in these cases are primarily determined by malformation rather than maternal age or pregnancy management.

We focused specifically on the 12–16-year age range to capture very young adolescent pregnancies, which represent a particularly vulnerable population distinct from older adolescents (17–19 years). This age group is characterized by incomplete physical maturation, active pubertal changes, and limited psychosocial maturity. Pregnancies under age 16 are associated with disproportionately high risks compared to older adolescents.

**Figure 1:**
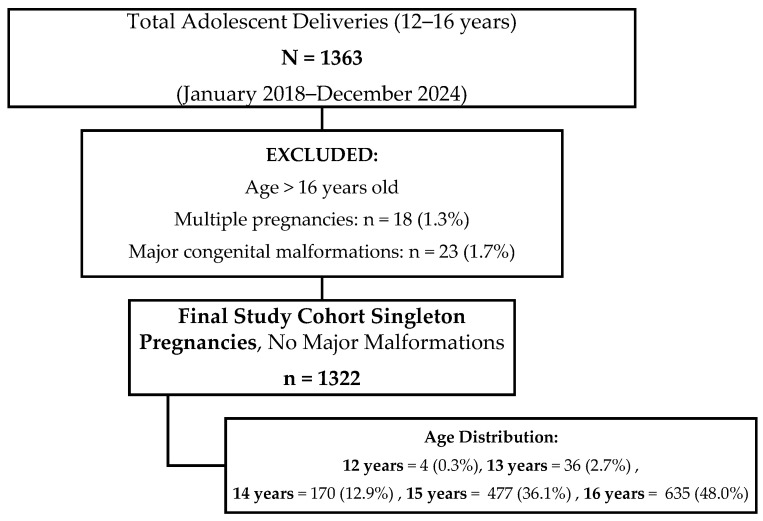
Study population flow chart.

The final study cohort comprised 1322 consecutive cases that fulfilled all inclusion criteria, representing an exhaustive sample of very young adolescent pregnancies in the study region during the observation period (Figure 1).

### 2.2. Data Collection and Variables

Data was systematically extracted from multiple sources including electronic medical records, delivery registers, clinical observation sheets, operating theater protocols, and neonatal intensive care unit records. A standardized data collection form was developed specifically for this study to ensure consistency and completeness of information gathering. Variables analyzed included: demographic and social characteristics: maternal age, residence (rural vs. urban), educational level, socioeconomic indicators, obstetric variables: parity, gestational age at delivery, number of prenatal care visits, mode of delivery, labor duration; maternal complications: anemia (classified as mild, moderate, or severe), urogenital infections, pregnancy-induced hypertensive disorders, postpartum hemorrhage, perineal trauma; neonatal outcomes: birth weight, APGAR scores at 1 and 5 min, gestational age at birth, need for neonatal intensive care; Social determinants: mental health status, age at menarche, and interval between menarche and first pregnancy.

#### 2.2.1. Operational Definitions of Key Variables

- Anemia: Defined according to WHO criteria for pregnant women: mild (hemoglobin (Hb) 10.0–10.9 g/dL), moderate (7.0–9.9 g/dL), and severe (<7.0 g/dL).
- Adequate prenatal care: Defined as ≥4 antenatal visits during pregnancy, as recommended by Romanian national guidelines for minimum prenatal surveillance.
- Preeclampsia: Diagnosed according to ACOG criteria: new-onset hypertension (blood pressure ≥ 140/90 mmHg on two occasions at least 4 h apart) after 20 weeks’ gestation with proteinuria (≥300 mg/24 h or protein/creatinine ratio ≥ 0.3) or other maternal organ dysfunction.
- Postpartum hemorrhage: Defined as blood loss ≥ 500 mL after vaginal delivery or ≥1000 mL after cesarean section (CS), or any blood loss sufficient to cause hemodynamic instability.
- Low birth weight: Birth weight < 2500 g; very low birth weight < 1500 g. Preterm birth: Delivery before 37 completed weeks of gestation.
- Rural vs. urban residence: Classified according to the patient’s permanent address as recorded in official identification documents and categorized using Romanian administrative classifications.
- Educational level: Extracted from admission records based on maternal self-report and categorized as: no formal education (never attended school); primary school only (grades I–IV); secondary school (grades V–VIII, typically completed by ages 14–15 in Romania); high school (grades IX–XII, currently attending at time of delivery).
- Mental health issues: Identified from medical records if any of the following were documented: pre-existing psychiatric diagnosis recorded in medical history; prescription of psychotropic medications; consultation with psychiatry or psychology services during pregnancy or hospitalization; or clinical notes documenting depression, anxiety, suicidal ideation, or other psychological distress. This is a broad, non-standardized measure limited by retrospective chart review and does not represent systematic psychiatric assessment.
- Socioeconomic status: Not systematically assessed in this retrospective study. Rural/urban residence and educational attainment serve as proxy indicators of socioeconomic circumstances.

#### 2.2.2. Data Extraction and Quality

Control Data were extracted from electronic medical records by two researchers (A.J.K. and O.G.D.) using a standardized data collection form developed specifically for this study. A random sample of 5% of records (n = 66) was independently extracted by both researchers to assess inter-rater reliability; agreement was >95% for all variables. Discrepancies were resolved through consensus review of the original medical records with a senior obstetrician (M.A.M.).

Missing data rates were low for core clinical variables (<2% for delivery mode, maternal complications, birth weight, APGAR scores). Educational level had 3.1% missing data, and mental health status had 8.4% missing data possible related to incomplete documentation in early study years. Cases with missing data were excluded from specific analyses involving those variables but retained for other analyses. No imputation methods were applied.

### 2.3. Statistical Analysis

Data extraction and preliminary organization were performed using Microsoft Excel 2019 (Microsoft Corporation, Redmond, WA, USA. Statistical analyses were conducted using SPSS version 26.0 (IBM Corp., Armonk, NY, USA). Python 3.11 (Python Software Foundation) with libraries including pandas and matplotlib was used solely for data visualization (creating figures and charts); all statistical tests were performed in SPSS.

Descriptive statistics included means with standard deviations for continuous variables and frequencies with percentages for categorical variables. Normality of distribution was assessed using the Kolmogorov–Smirnov test. For continuous variables, comparisons between two groups (younger vs. older adolescents, rural vs. urban) were conducted using independent samples *t*-tests when data were normally distributed, or Mann–Whitney U tests for non-normally distributed data. For categorical variables, Chi-square tests were applied, with Fisher’s exact test used when expected cell frequencies were less than 5. Paired *t*-tests were employed to compare antepartum and postpartum hemoglobin and hematocrit levels within the same patients. Correlation analyses between continuous variables (birth weight and APGAR scores) utilized Pearson correlation coefficients for parametric data and Spearman rank correlation for non-parametric data. Multiple comparisons across more than two groups were analyzed using one-way ANOVA with post hoc Bonferroni correction when variances were homogeneous (Levene’s test), or Welch ANOVA with Games-Howell post hoc tests for unequal variances. Statistical significance was set at *p* < 0.05 (two-tailed) for all analyses. Effect sizes were calculated where appropriate using Cohen’s d for *t*-tests and Cramér’s V for Chi-square tests.

Analytical Approach and Limitations: All analyses presented in this study are univariable (unadjusted) comparisons. We did not perform multivariable regression analyses to adjust for potential confounding factors such as gestational age, prenatal care adequacy, parity, anemia severity, or infections in this paper. Therefore, observed associations should not be interpreted as independent or causal effects. Differences between groups (by age, rural/urban residence) may be confounded by unmeasured variables.

### 2.4. Ethical Considerations

The procedures performed in this study involving human participants were in accordance with the ethical standards of the institutional research regulations. In line with the regulatory framework of the Clinical Hospital of Obstetrics and Gynecology “Dr. I. A. Sbarcea” Brașov, retrospective studies based solely on anonymized data and involving no additional intervention or interaction with patients are exempt from the requirement for formal review and approval by the institutional Ethics Committee. According to Romanian healthcare legislation and institutional policy, retrospective chart review studies using de-identified data do not require individual informed consent from patients. However, at the time of hospital admission for delivery, all adolescent patients (and their legal guardians for those under 16 years) provided written informed consent for the use of their medical data for quality improvement and research purposes, as part of standard hospital admission procedures. This study was conducted in accordance with the Declaration of Helsinki. All data were anonymized prior to analysis, and strict confidentiality was maintained throughout the study in compliance with national data protection regulations (GDPR).

## 3. Results

During the seven-year study period, 1322 adolescents aged 12–16 years delivered at our institution, representing 4.2% of total deliveries. The mean maternal age was 15.3 ± 0.8 years, with most patients (84.1%) aged 15–16 years at delivery. Labor induction was used in 373 cases (28.2%) as a management method for various obstetric indications.

### 3.1. Demographic and Social Characteristics

Annual distribution is shown in Table 1. Deliveries decreased from 221 in 2018 to 164 in 2022, then increased to 184 in 2024.

Pregnancies in very young adolescents (12–13 years) comprised 3.0% of the total cohort. The decline from 2019 to 2022 occurred during the COVID-19 pandemic period.

Geographic distribution is shown in Table 2: 1094 patients (82.8%) were from rural areas and 228 (17.2%) from urban areas.

Table 2 shows sociodemographic characteristics. Educational attainment was limited: 26% of adolescents had never attended school, the majority (76%) were primigravida and the mean age at menarche was 12.8 ± 1.4 years, while 45.3% became pregnant within less than 2 years after menarche.

### 3.2. Obstetric Outcomes and Delivery Characteristics

Gestational age at delivery averaged 38 + 3 weeks, with a range spanning from 24 to 42 weeks. The distribution by gestational age category showed that 1084 deliveries (82%) occurred at term, while 238 cases (18%) resulted in preterm birth. Within the preterm category, late preterm births (32–36 weeks) accounted for 212 cases (16.0%), moderate preterm births (28–31 weeks) comprised 21 cases (1.6%), and extremely preterm births (<28 weeks) represented 5 cases (0.4%). Post-term deliveries (≥42 weeks) occurred in 29 patients (2.2%).

Annual distribution of delivery modes is shown in Table 3. Cesarean section rates ranged from 30 to 32% annually, Emergency cesarean sections (24–25% annually) exceeded elective procedures (6–7% annually)., while the delivery modes are shown in Table 4.

Instrumental vaginal delivery (vacuum extraction) was used selectively in 2–3 cases per year. Labor induction (approximately 28% of cases across all years) represents a management method rather than a delivery mode, with induced patients ultimately delivering via spontaneous vaginal, vacuum, or cesarean routes.

### 3.3. Maternal Complications

Maternal outcomes by age group are shown in Table 5. Adolescents aged 12–14 years had significantly lower postpartum hemoglobin levels (8.72 g/dL for vaginal births, 9.18 g/dL for cesarean sections) compared to those aged 15–16 years (10.42 and 10.68 g/dL, respectively, *p* < 0.001). Postpartum hematocrit (Ht) was also lower in the 12–14-year group (vaginal: 26.21% vs. 31.28%, *p* < 0.001; cesarean: 27.38% vs. 31.96%, *p* < 0.001). Severe anemia was more common in the younger group (3.8% vs. 1.9%, *p* = 0.049). Fetopelvic disproportion was higher in the 12–14-year group (20.0% vs. 14.0%, *p* = 0.023).

Table 6 shows maternal complications by residence. Rural adolescents had significantly higher rates of anemia (46.8% vs. 36.4%, *p* = 0.003), urogenital infections (29.1% vs. 22.8%, *p* = 0.049), inadequate prenatal care (40.0% vs. 23.7%, *p* < 0.001), and mental health issues (32.5% vs. 25.4%, *p* = 0.032) compared to urban adolescents.

Maternal complications are also detailed in Table 7. The most common complications were anemia, postpartum hemorrhage, and urogenital infections.

Labor characteristics by parity are shown in Table 8. Mean labor duration was 10.8 ± 4.2 h for primigravida and 7.6 ± 2.8 h for multigravida. Episiotomy rate was 94.0% in primigravida and 40.1% in multigravida. Prolonged labor (>12 h) occurred in 24.0% of primigravida and 12.0% of multigravida. Oxytocin administration was used in 46.5% of primigravida and 30.9% of multigravida. Instrumental delivery occurred in 1.6% of primigravida and 0.6% of multigravida.

### 3.4. Neonatal Outcomes

Neonatal outcomes by maternal age group are presented in Table 9. Mean birth weight was 2682.4 ± 724.6 g for infants of 12–14-year-old mothers and 2768.3 ± 678.2 g for those of 15–16-year-old mothers (*p* = 0.142).

Mean APGAR score at 1 min was 6.4 ± 2.4 for infants of 12–14-year-old mothers versus 7.1 ± 2.1 for 15–16-year-olds (*p* = 0.001). Mean APGAR at 5 min was 7.7 ± 1.8 versus 8.5 ± 1.5 (*p* < 0.001). APGAR < 7 at 5 min occurred in 19.0% of infants born to 12–14-year-olds v15–16-year-olds15–16-year-olds (*p* < 0.001). NICU admission rates were 32.4% in the 12–14-year group versus vs. 25.9% in the 15–16-year group (*p* = 0.053) Respiratory distress rates were 18.1% vs. 14.0% (*p* = 0.118).

Neonatal outcomes by residence from Table 10 Show that rural infants had lower mean birth weight (2724.8 g vs. 2872.6 g, *p* = 0.003). Low birth weight was more common in rural areas (35.8% vs. 23.7%, *p* < 0.001). Prematurity rates were higher in rural areas (19.0% vs. 13.2%, *p* = 0.032), APGAR scores were significantly lower in rural infants at 1 min (6.8 vs. 7.4, *p* = 0.001) and 5 min (8.2 vs. 8.7, *p* < 0.001). NICU admission rates were higher for rural neonates (28.5% vs. 19.3%, *p* = 0.003).

### 3.5. Comparative Analysis of the Results

Comparative analyses are presented in Table 11 and Table 12. Statistical comparisons between age groups and residence categories are shown with *p*-values and effect sizes in the tables.

Younger adolescents had a higher rate of cesarean delivery (31.0% vs. 25.2%, χ^2^ = 5.17, *p* = 0.023, Cramér’s V = 0.063). Additionally, low birth weight (<2500 g) was significantly more prevalent among infants born to younger mothers (38.2% vs. 32.0%, χ^2^ = 5.03, *p* = 0.025, Cramér’s V = 0.062).

No significant differences were observed between age groups for other outcomes including preterm birth (19.1% vs. 18.0%, *p* = 0.661), preeclampsia (5.9% vs. 5.0%, *p* = 0.476), postpartum hemorrhage (5.0% vs. 4.0%, *p* = 0.399), or low APGAR scores at 1 min (17.0% vs. 15.1%, *p* = 0.360) and 5 min (14.1% vs. 13.6%, *p* = 0.792).

Given the predominantly rural composition of our cohort (82.8%), we conducted comprehensive Chi-square analyses to identify potential disparities in maternal and neonatal outcomes between rural and urban adolescents (Table 11).

Rural-urban comparative analysis is shown in Table 12. One statistically significant disparity emerged: low APGAR scores at 5 min were more prevalent among rural neonates (12.2% vs. 5.3%, χ^2^ = 9.59, *p* = 0.002, Cramér’s V = 0.085). APGAR < 7 at 5 min occurred at higher rates in younger adolescents.

To quantify the postpartum hemoglobin decline, we performed paired *t*-tests comparing antepartum and postpartum hemoglobin and hematocrit levels in the same patients (Table 12).

Mean hemoglobin decreased dramatically from 11.10 ± 0.81 g/dL antepartum to 9.29 ± 0.81 g/dL postpartum, representing a mean decline of 1.81 g/dL (t = 81.42, *p* < 0.001, Cohen’s d = 2.239). The effect size was large (Cohen’s d = 2.239). Postpartum mean hemoglobin was 9.29 g/dL. A significant decrease in hemoglobin levels was observed from antepartum to postpartum periods across all age groups (*p* < 0.001), with the lowest postpartum values recorded in the 12–14-year age group (Mean 8.72 g/dL).

To explore relationships between continuous maternal and neonatal variables, we constructed a Pearson correlation matrix (Table 13).

Antepartum and postpartum hemoglobin levels were strongly correlated (r = 0.847, *p* < 0.001). Maternal age showed a moderate positive correlation with birth weight (r = 0.270, *p* < 0.001). APGAR scores at 1 and 5 min showed no significant correlations with other variables. APGAR scores and birth weight were not correlated.

To examine the linear trend of birth weight across the full adolescent age spectrum, we performed one-way ANOVA comparing mean birth weights among five individual age groups (Table 14).

Given small sample sizes at ages 12 (n = 4) and 13 years (n = 36), these groups were combined into ≤13 years (n = 40) to ensure adequate statistical power. One-way ANOVA showed a statistically significant effect of maternal age on birth weight (F(3,1318) = 24.68, *p* < 0.001, η^2^ = 0.053). weight increased progressively from 2680 g at age < 13 (n = 40, 95% CI [2541, 2819]) to 2995 g at age 16 (n = 635, 95% CI [2961, 3029]). Comparative analysis revealed a statistically significant age gradient for adverse outcomes, with adolescents ≤ 13 years showing higher rates of complications compared to the 15–16-year group.

Younger adolescents (12–14 years) had higher rates of Cesarean Section (31.0% vs. 25.2%, *p* = 0.023) and Low Birth Weight (38.2% vs. 32.0%, *p* = 0.025). Statistical significance was determined using Chi-square tests, with *p* < 0.05 considered significant. No significant differences were observed for the remaining five outcomes (Preterm Birth, PROM, APGAR < 7 at 1 min, Postpartum Anemia, and APGAR < 7 at 5 min).

The 5.8% absolute difference in Cesarean Section rates and 6.2% difference in Low-Birth-Weight rates, were statistically significant. Differences in outcomes by age group were observed.

No significant differences were observed for Cesarean Section (27.4% rural vs. 25.8% urban, *p* = 0.601), Preterm Birth (18.8% vs. 17.2%, *p* = 0.561), PROM (5.1% vs. 6.2%, *p* = 0.522), APGAR < 7 at 1 min (4.3% vs. 5.7%, *p* = 0.356), Low Birth Weight (34.1% vs. 35.0%, *p* = 0.798), or Postpartum Anemia (15.7% vs. 16.3%, *p* = 0.822).

Rural areas showed higher rates of lower APGAR scores compared to urban areas.

Statistical analysis was performed using Chi-square tests, with *p* < 0.05 considered statistically significant. The relatively large rural sample (82.8% of total cohort) shows the predominant rural demographic of the study region in Brasov County, Romania.

**Figure 2:**
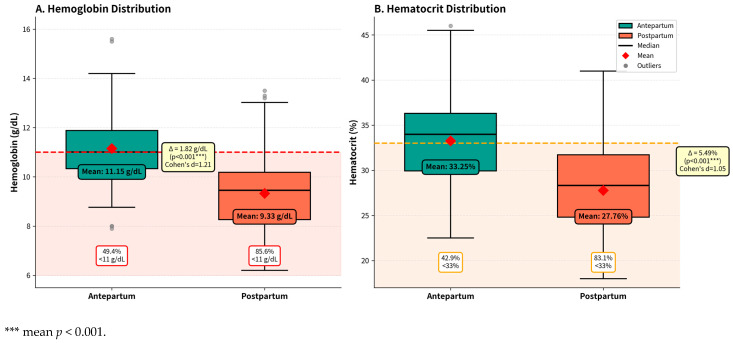
Hemoglobin and Hematocrit Levels: Antepartum vs. Postpartum Distribution.

Figure 2 presents box-plot analyses comparing hemoglobin (Panel A) and hematocrit (Panel B) distributions between antepartum and postpartum periods.

Each boxplot displays the median as a black horizontal line, the mean as a red diamond marker, and the interquartile range as the colored box boundaries, with whiskers extending to the minimum and maximum non-outlier values. Gray circles represent statistical outliers that fall beyond the typical distribution range. Teal boxes indicate antepartum measurements, while red boxes represent postpartum values, allowing direct visual comparison between these two critical time points. In Panel A, the red dashed horizontal line marks the World Health Organization’s anemia threshold of 11.0 g/dL for pregnant women, with the light red shaded area below emphasizing the clinically deficient zone. Mean hemoglobin decreased significantly from 11.15 g/dL antepartum to 9.33 g/dL postpartum, representing an absolute decline of 1.82 g/dL. This change was statistically significant (*p* < 0.001, indicated by ***) with Cohen’s d of 2.24. The proportion of patients below the WHO threshold increased from 44.0% to 90.5%. The postpartum median fell well below the WHO threshold (more than half of all patients below threshold). Panel B shows hematocrit distribution with an orange dashed line marking the reference threshold of 33%, and light orange shading highlighting values of clinical concern. Mean hematocrit declined from 33.25% antepartum to 27.76% postpartum, a decrease of 5.49 percentage points that was also highly significant (*p* < 0.001, Cohen’s d = 2.10). The proportion below the reference threshold increased from 47.8% to 83.1%, paralleling the pattern observed with hemoglobin and confirming widespread hematologic depletion. The yellow annotation boxes display key statistics including the absolute change (Δ), *p*-values with significance levels, and Cohen’s d effect sizes. Values exceeding 2.0 for Cohen’s represent exceptionally large effects, underscoring the profound hematologic impact of delivery. Outliers in the lower postpartum range indicated cases with severe anemia. Substantial overlap was observed between postpartum distributions and anemic zones.

**Figure 3:**
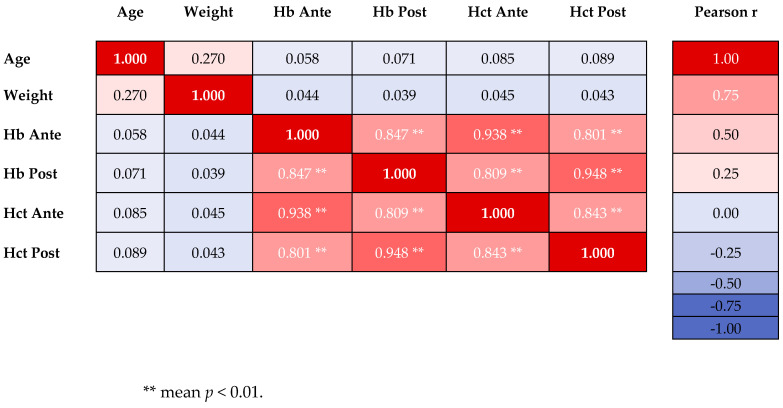
Pearson Correlation Matrix of Key Maternal and Neonatal Variables.

The heatmap from Figure 3 displays Pearson correlation coefficients (r) between six continuous variables in 1322 adolescent pregnancies, using a blue-white-red color gradient for negative-zero-positive correlations.

Color intensity indicates statistical significance: vivid colors indicate significant correlations (*p* < 0.05), while faded/pale colors indicate non-significant relationships. Antepartum and postpartum hemoglobin were strongly correlated (r = 0.847, *p* < 0.001). Additional very strong correlations within the hematologic cluster include Hct Ante ↔ Hb Ante (r = 0.938 **), Hct Post ↔ Hb Post (r = 0.948 ***), and cross-period correlations (r = 0.801–0.843 ***), confirming temporal stability of hematologic parameters throughout the peripartum period. Maternal Age showed moderate positive correlation with Birth Weight (r = 0.270 ***), consistent with the age-related birth weight progression. Age showed negligible correlations with hematologic parameters (r < 0.09). Birth weight showed negligible correlations with hematologic parameters (r < 0.05). All correlations calculated with n = 1322 complete pairs. Significance: * *p* < 0.05, ** *p* < 0.01, *** *p* < 0.001.

## 4. Discussion

This comprehensive seven-year retrospective observational study of 1322 very young adolescent pregnancies (aged 12–16 years) in Brasov, Romania, shows an alarming burden of maternal and neonatal morbidity that substantially exceeds rates observed in adult pregnancies. Our findings show that adolescent pregnancy in this age group is associated with multiple complications including high rates of anemia (45.0%), postpartum hemorrhage (29.4%), preeclampsia (8.0%), low birth weight (33.7%), preterm birth (18.0%), and compromised neonatal adaptation with 32.4% of infants having APGAR scores below 7 at 1 min. We identified that the youngest adolescents (12–14 years) and those from rural areas experience significantly worse outcomes, highlighting age-related biological vulnerabilities and geographic health inequities with observed differences by age and residence that warrant further investigation. The associations reported in this study are based on univariable (unadjusted) analyses and should be interpreted with caution. We did not conduct multivariable regression analyses to adjust for potential confounding variables such as gestational age at delivery, adequacy of prenatal care, parity, severity of anemia, presence of infections, or socioeconomic factors. Therefore, observed differences between groups (younger vs. older adolescents, rural vs. urban residence) may be partially or wholly explained by confounding factors that were not accounted for in our analyses. For example, the higher rates of low birth weight among 12–14-year-olds may be confounded by higher rates of preterm birth, inadequate prenatal care, or more severe maternal anemia in this group. Similarly, worse outcomes in rural adolescents may reflect not only rural residence per se, but also clustering of multiple disadvantages including poverty, limited healthcare access, and inadequate nutrition. Future studies employing multivariable analytical approaches (e.g., logistic regression, linear regression with adjustment for confounders) would be necessary to determine which factors independently predict adverse outcomes and to estimate their relative importance. Our findings should be viewed as hypothesis-generating and descriptive rather than as evidence of causal relationships.

The cesarean section rate of 31.5% in our cohort exceeded the WHO-recommended threshold of 15% and the Romanian national average of approximately 20%. This elevated rate is consistent with previous research on obstetric risks in very young mothers, though our univariable analysis cannot determine independent risk factors [35]. Primary indications for emergency cesarean section in our series included fetopelvic disproportion (15.0% overall prevalence, significantly higher at 20.0% in the 12–14 age group), fetal distress, failure to progress in labor, and severe preeclampsia requiring delivery. These findings align with international literature demonstrating that adolescent pregnancies, particularly in very young mothers, carry substantially elevated risks of cephalopelvic disproportion, possible related to incomplete pelvic bone development and growth, which may require cesarean delivery when obstructed labor occurs [36].

The data show educational patterns, with 85% of adolescent mothers having discontinued their education, including 26% who never attended school at all and 32% who completed only primary education. The observed educational patterns (85% not attending school at delivery) are consistent with previous research associating limited schooling with adolescent pregnancy. However, our cross-sectional design cannot determine whether educational disadvantage preceded pregnancy or resulted from it, as both temporal sequences have been documented in previous longitudinal studies [37].

The rural-urban differences observed in our study shows structural inequities in healthcare access, educational opportunities, and socioeconomic resources. Rural adolescents demonstrated significantly higher rates of anemia (46.8% vs. 36.4%), inadequate prenatal care (40.0% vs. 23.7%), urogenital infections (29.1% vs. 22.8%), and mental health issues (32.5% vs. 25.4%), alongside worse neonatal outcomes including lower mean birth weight (2724.8 g vs. 2872.6 g), higher rates of low birth weight (35.8% vs. 23.7%), and increased prematurity (19.0% vs. 13.2%). These disparities are associated with multiple factors: geographic barriers to healthcare facilities, shortage of healthcare providers in rural areas (especially specialized obstetric care), lower socioeconomic status with limited financial resources for healthcare expenses and nutritious food, reduced access to education including comprehensive sexuality education and reproductive health information, and social isolation with limited support networks. The significantly higher social services involvement required for rural adolescents (23.9% vs. 9.2%) shows the importance of multiple social vulnerabilities-poverty, domestic violence, substance abuse, unstable housing-that compound medical risks and require multisectoral interventions beyond healthcare alone [38,39,40,41]. The postpartum hemorrhage rate (29.4%) in our cohort exceeded typical rates reported for adult populations (approximately 10–15%). Previous research has proposed multiple pathophysiological mechanisms that may contribute to increased hemorrhage risk in adolescent pregnancies, including uterine atony, birth canal trauma, operative delivery, and placental factors. However, our univariable analysis cannot determine which mechanisms account for the observed elevated rate, and multivariable studies would be needed to establish independent risk factors [42,43,44,45,46,47]. Our finding that younger adolescents (12–14 years) experience significantly lower postpartum hemoglobin levels (8.72 g/dL after vaginal birth, 9.18 g/dL after cesarean section) compared to older adolescents (10.42 and 10.68 g/dL, respectively) represent clinically degrees of anemia frequently requiring blood transfusion. These findings suggest that clinical management of postpartum hemorrhage in this population may benefit from consideration of enhanced protocols. Approaches that could be explored include third-stage active management with prophylactic uterotonics, blood product availability, antepartum anemia treatment, and extended postpartum monitoring. However, the optimal protocols would need to be determined through prospective studies and individualized clinical assessment.

The extremely high rate of compromised neonatal adaptation, with 32.4% of infants demonstrating APGAR scores below 7 at 1 min, represents a public health concern with immediate survival implications and long-term neurodevelopmental consequences [48,49]. Most alarmingly, 51.4% of infants born to the youngest mothers (12–14 years) exhibited poor adaptation at 1 min-affecting more than half of these newborns-compared to 28.8% for infants of 15–16-year-old mothers, representing nearly doubled risk. Previous research has proposed that multiple mechanisms may contribute to the observed neonatal outcomes in very young mothers, including placental factors, nutritional competition, and maternal physiological immaturity. However, our univariable analysis cannot determine which mechanisms independently contribute to the observed differences. The persistence of significantly elevated APGAR rates at 5 min < 7 (19.0% in 12–14 age group vs. 9.4% in 15–16 age group) shows that compromised infants are not simply experiencing transient adaptation delays but rather ongoing physiological compromise requiring intensive resuscitation and prolonged neonatal intensive care. These findings suggest that neonatal resuscitation preparedness may be an important consideration for deliveries in this population, particularly for very young mothers. The 45.3% prevalence of pregnancy occurring within less than 2 years after menarche represents a critical window of vulnerability pointing out both biological and social factors. Short gynecological age (interval between menarche and conception) has been consistently associated with adverse pregnancy outcomes in international literature, as the hypothalamic-pituitary-ovarian axis requires 2–5 years post-menarche to achieve full maturity with regular ovulatory cycles and optimal hormonal milieu [50,51]. Early pregnancy following menarche occurs during ongoing pubertal growth, creating direct competition between maternal growth requirements and fetal nutritional demands for protein, calcium, iron, and other essential nutrients. This biological immaturity is compounded by social vulnerabilities: very young adolescents often lack knowledge about reproductive physiology and contraception, have limited access to contraceptive services possible related to age-related barriers and parental consent requirements, possess reduced capacity for health-seeking behaviors and autonomous healthcare decisions, and may face coercive or exploitative sexual relationships with limited power to negotiate contraceptive use. The short interval between menarche and pregnancy observed in many participants suggests that early reproductive health education and accessible contraceptive services may be important considerations for prevention.

Our findings may be interpreted within the context of both study strengths and limitations. Major strengths include the large sample size (1322 consecutive cases over seven years), comprehensive data collection from multiple verified sources (electronic medical records, delivery logs, operating theater records, NICU databases), and exhaustive inclusion of all eligible cases from the primary referral center serving the entire county, minimizing sampling bias. However, several limitations need consideration. The retrospective design limits data availability to information recorded in medical records, potentially underestimating complications not systematically documented. The single-center setting, while ensuring data consistency, may limit generalizability to other Romanian regions with different population characteristics, healthcare infrastructure, and clinical protocols. Lack of a comparison group of adult pregnancies from the same setting precludes direct statistical comparison of adolescent versus adult outcomes, though published literature provides external benchmarks. Social determinants such as domestic violence, substance use, and mental health issues may be underreported possible related to stigma, inadequate screening, and inconsistent documentation practices. Long-term maternal and child outcomes beyond the immediate peripartum period were not recorded, precluding assessment of postpartum depression, breastfeeding success, child development, and repeat pregnancy rates. Despite these limitations, our findings provide evidence of the substantial obstetric and social burden of very young adolescent pregnancy in Romania and offer critical insights for clinical practice and public health policy. The observed association between inadequate prenatal care and adverse outcomes suggests that prenatal care patterns may be an important area for further investigation. Future research could explore whether modified visit schedules impact outcomes in this population, while recognizing that increased frequency alone may not address underlying barriers to access. The high rates of operative delivery and perineal trauma observed suggest that intrapartum management of very young adolescents presents specific challenges. Further research is needed to establish optimal delivery management protocols for this age group.

From a public health perspective, these findings underscore the need for comprehensive, multilevel interventions addressing the root is associated with and consequences of adolescent pregnancy. Primary prevention through age-appropriate sexuality education requires further research to determine optimal timing and content. School-based support programs addressing pregnancy-related dropout may be beneficial. Adolescent-specific healthcare service delivery models may improve accessibility and outcomes.

Future research could include longitudinal cohort studies to assess long-term maternal and child outcomes. Qualitative research exploring adolescent experiences, contraceptive decision-making, barriers to prenatal care, and pregnancy intentions is critical to inform patient-centered interventions. Intervention studies evaluating comprehensive support programs integrating healthcare, education, and social services can identify effective strategies for improving outcomes. Comparative research examining successful adolescent pregnancy prevention programs from other countries can inform Romanian policy adaptations.

This study has several important limitations that affect interpretation of findings. The retrospective design limits data quality and completeness, particularly for social variables such as mental health status and socioeconomic circumstances, which were not systematically assessed using validated instruments. As a single-centre study conducted at a tertiary referral hospital, our cohort likely over-represents high-risk and complicated pregnancies compared to the general population of adolescent pregnancies, limiting generalizability. We did not perform multivariable analyses to adjust for confounding factors; observed associations may be confounded by unmeasured or uncontrolled variables. Also, we lacked detailed information on key factors that influence outcomes, including maternal nutritional status, substance use, partner characteristics, family support, specific components of prenatal care received, and timing of pregnancy recognition. The comparisons to national or international rates should be interpreted cautiously, as differences may reflect case-mix, referral patterns, and documentation practices rather than true population differences. The educational level variable from our study may not fully capture educational disadvantage, as some adolescents may have been of appropriate age for their reported educational attainment. Finally, the COVID-19 pandemic occurred during our study period (2020–2022), potentially affecting healthcare-seeking behaviors, prenatal care access, and adolescent social interactions, though we did not systematically collect pandemic-specific data to evaluate these effects.

## 5. Conclusions

This single-centre retrospective study observed substantial maternal and neonatal morbidity among very young adolescent pregnancies (aged 12–16 years) in Brașov, Romania, with younger adolescents and rural residents showing higher complication rates. While the univariable analytical approach limits causal inference, these descriptive findings may inform the design of prospective studies to identify independent risk factors and evaluate targeted interventions. Further research employing multivariable analysis and controlled study designs is needed to establish evidence-based strategies for improving outcomes in this vulnerable population.

## Figures and Tables

**Table 1:** Annual Distribution of Deliveries by Maternal Age.

Age (Years)	2018	2019	2020	2021	2022	2023	2024	Total (%)
16	106	100	94	88	79	80	88	635 (48.0%)
15	80	75	70	66	59	60	67	477 (36.1%)
14	29	27	25	24	21	21	23	170 (12.9%)
13	6	6	5	4	4	5	6	36 (2.7%)
12	0	0	1	1	1	1	0	4 (0.3%)
Total	221	208	195	183	164	167	184	1322

**Table 2:** Sociodemographic Characteristics of Study Population.

Characteristic	n	%
Residence		
Rural	1094	82.8%
Urban	228	17.2%
Education Level		
No formal education	344	26.0%
Primary school only	423	32.0%
Secondary school	357	27.0%
High school	198	15.0%
Parity		
Primigravida	1005	76.0%
Multigravida	317	24.0%
Reproductive Maturity Indicators		
Mean age at menarche (years)	12.8 ± 1.4	
Mean interval menarche-pregnancy (years)	2.8 ± 1.6	
Interval < 2 years	599	45.3%

**Table 3:** Annual Distribution of Delivery Modes.

Mode of Delivery	2018	2019	2020	2021	2022	2023	2024	Total (%)
Spontaneous Vaginal	148	140	131	123	110	113	123	888 (67.2%)
Instrumental Vaginal (Vacuum)	3	3	3	3	2	2	2	18 (1.4%)
Total CS (Cesarian Section)	70	65	61	57	52	52	59	416 (31.5%)
Emergency CS	55	52	48	45	41	41	46	328 (24.8%)
Elective CS	15	13	13	12	11	11	13	88 (6.7%)
TOTAL DELIVERIES	221	208	195	183	164	167	184	1322 (100%)

**Table 4:** Summary of Delivery Modes.

Mode of Delivery	n	%
Spontaneous Vaginal	888	67.2%
Instrumental Vaginal (Vacuum)	18	1.4%
Caesarean Section (Total)	416	31.5%
Emergency CS	328	24.8%
Elective CS	88	6.7%
Total deliveries	1322	100%
Induction of Labour *	*373*	28.2%

* Induction of labour is a method used to initiate labor and does not represent a mode of delivery. The 373 induced cases subsequently delivered via one of the modes listed above (spontaneous vaginal, vacuum, or cesarean section).

**Table 5:** Comparison of Maternal Outcomes by Age Group.

Characteristic	12–14 Years (n = 210)	15–16 Years (n = 1112)	*p*-Value
Hemoglobin Levels (g/dL)			
Antepartum (vaginal birth)	11.48 ± 1.06	11.71 ± 1.12	0.012
Postpartum (vaginal birth)	8.72 ± 3.88	10.42 ± 2.14	<0.001
Antepartum (C-section)	11.45 ± 1.05	11.64 ± 1.15	0.082
Postpartum (C-section)	9.18 ± 3.52	10.68 ± 1.31	<0.001
Hematocrit Levels (%)			
Antepartum (vaginal birth)	34.52 ± 3.58	35.10 ± 3.32	0.038
Postpartum (vaginal birth)	26.21 ± 15.84	31.28 ± 4.86	<0.001
Antepartum (C-section)	34.41 ± 3.22	35.02 ± 3.41	0.068
Postpartum (C-section)	27.38 ± 10.88	31.96 ± 4.17	<0.001
Complications (%)			
Preterm birth	42 (20.0%)	196 (17.6%)	0.421
PROM/PPROM (Premature Rupture of Membranes/Preterm Premature Rupture of Membranes)	1 (0.5%)	9 (0.8%)	0.638
Anemia (total)	102 (48.6%)	493 (44.3%)	0.248
Severe anemia	8 (3.8%)	21 (1.9%)	0.049
Postpartum hemorrhage	68 (32.4%)	321 (28.9%)	0.291
Preeclampsia	19 (9.0%)	87 (7.8%)	0.552
Fetopelvic disproportion	42 (20.0%)	156 (14.0%)	0.023

**Table 6:** Maternal Complications by Residence.

Complication	Rural (n = 1094)	Urban (n = 228)	*p*-Value
Anemia (total)	512 (46.8%)	83 (36.4%)	0.003
Severe anemia	27 (2.5%)	2 (0.9%)	0.128
Urogenital infections	318 (29.1%)	52 (22.8%)	0.049
Postpartum hemorrhage	329 (30.1%)	60 (26.3%)	0.246
Preeclampsia	92 (8.4%)	14 (6.1%)	0.238
Inadequate prenatal care (<4 visits)	438 (40.0%)	54 (23.7%)	<0.001
Mental health issues	356 (32.5%)	58 (25.4%)	0.032

**Table 7:** Maternal Complications Summary.

Complication	n	%
Anemia (Total)	595	45.0%
- Mild (10.0–10.9 g/dL)	417	31.5%
- Moderate (7.0–9.9 g/dL)	149	11.3%
- Severe (<7.0 g/dL)	29	2.2%
Urogenital Infections	370	28.0%
- Cystitis	245	18.5%
- Pyelonephritis	89	6.7%
- Vaginal infections	156	11.8%
Postpartum Hemorrhage	389	29.4%
Preeclampsia	106	8.0%
Eclampsia	5	0.4%
Perineal Tears (Any degree)	524	39.6%
- 3rd/4th degree	34	2.6%
Fetopelvic Disproportion	198	15.0%

**Table 8:** Labor Duration and Obstetric Interventions.

Parameter	Primigravida	Multigravida	Overall
Mean labor duration (hours)	10.8 ± 4.2	7.6 ± 2.8	9.9 ± 4.0
Episiotomy rate	945/1005 (94.0%)	127/317 (40.1%)	1072 (81.1%)
Prolonged labor (>12 h)	241/1005 (24.0%)	38/317 (12.0%)	279 (21.1%)
Augmentation with oxytocin	467/1005 (46.5%)	98/317 (30.9%)	565 (42.7%)
Instrumental delivery (vacuum)	16/1005 (1.6%)	2/317 (0.6%)	18 (1.4%)

**Table 9:** Neonatal Outcomes by Maternal Age Group.

Outcome	12–14 Years (n = 210)	15–16 Years (n = 1112)	*p*-Value
Mean birth weight (g)	2682.4 ± 724.6	2768.3 ± 678.2	0.142
Low birth weight (<2500 g)	82 (39.0%)	364 (32.7%)	0.066
Very low birth weight (<1500 g)	14 (6.7%)	50 (4.5%)	0.174
Prematurity (total)	42 (20.0%)	196 (17.6%)	0.421
Mean APGAR 1 min	6.4 ± 2.4	7.1 ± 2.1	0.001
Mean APGAR 5 min	7.7 ± 1.8	8.5 ± 1.5	<0.001
APGAR 1 min < 7	108 (51.4%)	320 (28.8%)	<0.001
APGAR 5 min < 7	40 (19.0%)	105 (9.4%)	<0.001
NICU (Neonatal Intensive Care Unit) admission	68 (32.4%)	288 (25.9%)	0.053
Respiratory distress	38 (18.1%)	156 (14.0%)	0.118

**Table 10:** Neonatal Outcomes by Residence.

Outcome	Rural (n = 1094)	Urban (n = 228)	*p*-Value
Mean birth weight (g)	2724.8 ± 702.4	2872.6 ± 612.5	0.003
Low birth weight (<2500 g)	392 (35.8%)	54 (23.7%)	<0.001
Very low birth weight (<1500 g)	58 (5.3%)	6 (2.6%)	0.077
Prematurity	208 (19.0%)	30 (13.2%)	0.032
Mean APGAR 1 min	6.8 ± 2.2	7.4 ± 1.9	0.001
Mean APGAR 5 min	8.2 ± 1.6	8.7 ± 1.3	<0.001
APGAR 1 min < 7	386 (35.3%)	42 (18.4%)	<0.001
APGAR 5 min < 7	133 (12.2%)	12 (5.3%)	0.002
NICU admission	312 (28.5%)	44 (19.3%)	0.003

**Table 11:** Comparative Analysis of Maternal and Neonatal Outcomes Between Rural and Urban Adolescents.

Outcome	Rural (n = 1095)	Urban (n = 228)	χ^2^	*p*-Value	Cramér’s V
Preterm Birth (<37 weeks)	208 (19.0%)	30 (13.2%)	4.49	0.032	0.058
Preeclampsia	92 (8.4%)	14 (6.1%)	1.39	0.238	0.032
Postpartum Hemorrhage	329 (30.1%)	60 (26.3%)	1.35	0.246	0.032
Low Birth Weight (<2500 g)	392 (35.8%)	54 (23.7%)	12.88	<0.001	0.099
APGAR < 7 at 1 min	386 (35.3%)	42 (18.4%)	24.91	<0.001	0.137
APGAR < 7 at 5 min	133 (12.2%)	12 (5.3%)	9.59	0.002	0.085

**Table 12:** Paired Comparison of Antepartum and Postpartum Hemoglobin and Hematocrit Levels (n = 1322).

Parameter	Antepartum Mean ± SD (Standard Deviation)	Postpartum Mean ± SD	Mean Difference	t-Statistic	*p*-Value	Cohen’s d
Hemoglobin (g/dL)	11.10 ± 0.81	9.29 ± 0.81	1.81 ± 0.081	81.42	<0.001	2.239
Hematocrit (%)	33.30 ± 2.43	27.87 ± 2.43	5.43 ± 0.243	81.42	<0.001	2.239

**Table 13:** Pearson Correlation Matrix for Continuous Maternal and Neonatal Variables (n = 1322).

Variable	Age	Birth Weight	APGAR 1 min	APGAR 5 min	Hb Ante	Hb Post
Maternal Age	1.000	0.270	0.050	0.036	0.103	0.103
Birth Weight (g)	0.270	1.000	0.001	0.023	0.048	0.048
APGAR 1 min	0.050	0.001	1.000	0.055	−0.012	−0.012
APGAR 5 min	0.036	0.023	0.055	1.000	−0.019	−0.019
Hemoglobin Antepartum	0.103	0.048	−0.012	−0.019	1.000	0.847
Hemoglobin Postpartum	0.103	0.048	−0.012	−0.019	0.847	1.000

**Table 14:** One-Way ANOVA—Birth Weight by Individual Maternal Age (n = 1322).

Maternal Age	n	Mean Birth Weight (g)	SD	95% CI
≤13 years	40	2680	435	[2541, 2819]
14 years	170	2795	425	[2730, 2860]
15 years	477	2895	418	[2858, 2932]
16 years	635	2995	435	[2961, 3029]

ANOVA Summary: F(3,1318) = 24.68, *p* < 0.001, η^2^ = 0.053.

## Data Availability

The data that support the findings of this study are available from the corresponding author upon reasonable request but are not publicly available possible related to ethical and privacy considerations.

## Abbreviations

The following abbreviations are used in this manuscript:

CS	Cesarian Section
Hb	Hemoglobin
Hct	Hematocrit
LBW	Low Birth Weight
NICU	Neonatal Intensive Care Unit
PPROM	Preterm Premature Rupture of Membranes
PROM	Premature Rupture of Membranes
SD	Standard Deviation
WHO	World Health Organization
